# Identification of a histone family gene signature for predicting the prognosis of cervical cancer patients

**DOI:** 10.1038/s41598-017-16472-5

**Published:** 2017-11-28

**Authors:** Xiaofang Li, Run Tian, Hugh Gao, Yongkang Yang, Bryan R. G. Williams, Michael P. Gantier, Nigel A. J. McMillan, Dakang Xu, Yiqun Hu, Yan’e Gao

**Affiliations:** 1 Department of Obstetrics and Gynecology, the Second Affiliated Hospital, Xi’an Jiaotong University, Xi’an, P.R. China; 2 Department of Orthopaedics, the Second Affiliated Hospital, Xi’an Jiaotong University, Xi’an, P.R. China; 3 Hudson Institute of Medical Research, Clayton, Victoria, Australia; 4 Department of Molecular and Translational Science, Monash University, Clayton, Victoria, Australia; 5 Menzies Health Institute Queensland, Griffith University, Gold Coast, Australia; 6 Faculty of Medical Laboratory Science, Ruijin Hospital, Shanghai Jiaotong University School of Medicine, Shanghai, China

## Abstract

Heterogeneity in terms of tumor characteristics, prognosis, and survival among cancer patients is an unsolved issue. Here, we systematically analyzed the aberrant expression patterns of cervical cancer using RNA-Seq data from The Cancer Genome Atlas (TCGA). We incorporated gene profiling, molecular signatures, functional and pathway information with gene set enrichment and protein-protein interaction (PPI) network analysis, to identify sub-networks of genes. Those identified genes relating to DNA replication and DNA repair-mediated signaling pathways associated with systemic lupus erythematosus (SLE). Next, we combined cross-validated prognostic scores to build an integrated prognostic model for survival prediction. The combined approach revealed that the DNA repair-mediated including the functional interaction module of 18 histone genes (Histone cluster 1 H2A, B and H4), were significantly correlated with the survival rate. Furthermore, five of these histone genes were highly expressed in three cervical cancer cohorts from the Oncomine database. Comparison of high and low histone variant-expressing human cervical cancer cell lines revealed different responses to DNA damage, suggesting protective functions of histone genes against DNA damage. Collectively, we provide evidence that two SLE-associated gene sets (HIST1H2BD and HIST1H2BJ; and HIST1H2BD, HIST1H2BJ, HIST1H2BH, HIST1H2AM and HIST1H4K) can be used as prognostic factors for survival prediction among cervical cancer patients.

## Introduction

Human cervical cancer (HCC) is the fourth most frequently diagnosed cancer and is associated with high cancer-related mortality in women^1^. The widespread practice of cervical cytologic screening (termed the Papanicolaou test) has substantially reduced HCC mortality. The greatest risk factor for HCC is infection with certain types of the human papillomavirus (HPV), but viral infection alone is not sufficient for its development^2^. The pathogenesis of cervical cancer is still unclear and probably involves the aberrant expression of numerous oncogenes and tumor suppressors. Although radical surgery and radiotherapy represent effective treatment modalities, 30% of patients will still develop progressive or recurrent tumors, with the pelvis being the most common site of failure^3^. The ability to predict which patients are at a high risk of recurrence may allow for the development of novel therapeutic strategies limiting such recurrence. Although common clinic-pathological parameters (histological grade, stage and several other biomarkers) have been employed for recurrence prediction, they are characterized by insufficient sensitivity and specificity^4^. Thus, the identification of novel markers to increase the power of prediction of prognosis or cancer development among patients with cervical carcinoma is urgently needed.

The genetic abnormalities that drive tumorigenesis are often coupled with epigenetic alterations, such as aberrant histone modifications, which may help oncogenic drivers accelerate cancer progression, metastasis, and therapy resistance^5^. In the genome, negatively charged, linear DNA is highly compacted and organized into three-dimensional (3D) chromosomes. DNA is coiled around histones (the main proteins of chromosomes) to form nucleosomes, the basic structural units of chromosomes. Histones are positively charged in the N-terminus with abundant lysine and arginine residues and can thus bind tightly to DNA to constrain its accessibility. Histone modification proteins including histone family genes, histones H2A, H2B, H3 and H4; two heterodimers of H2A/H2B; and one H3/H4 tetramer associated with DNA, form the compact structure of chromatin in nucleosomes. These histones can be modified by a variety of enzymes. H2A/H2B play important roles in processes on the chromatin that allow for transcription, DNA replication and DNA repair. Mono-ubiquitination of histone H2B at lysine 120 (H2Bub1) by the ubiquitin ligases RNF20/40, is essential for proper DNA repair. Loss of H2Bub1 results in increased H2AX phosphorylation and a prolonged DNA damage response^6,7^. In mammals, mono-ubiquitinated H2B is associated with the transcribed regions of active genes^8^. Interestingly, the RNF20/40 complex has also been implicated in tumorigenesis. The tumor suppressor function of H2Bub1 was also supported by a recent study demonstrating that a decrease in H2Bub1 levels strongly correlates with breast cancer progression, indicating a role for H2Bub1 during tumorigenesis and DNA repair^9^. The preliminary studies suggest that histone genes are involved in a number of human cancers, but a comprehensive analysis of the gene family, which may be prognostic biomarkers, has not been performed.

In the present study, we investigated the differences in mRNA expression between tumor and normal tissues in multiple cervical cancers using TCGA and Oncomine databases, and identified the histone family gene signature by integrating gene profiling, molecular signatures and functional and pathway information with gene set enrichment analysis and protein-protein interaction (PPI) network analysis. Additionally, histone genes expression was validated in cervical cancer cell lines, and a DNA repair function assay showed that a subset of histone genes has an important impact on tumor phenotypes. The prognostic significance of these histone variants was also determined via the Kaplan-Meier Plotter (KM Plotter).

## Materials and Methods

### Datasets

The Cancer Genome Atlas (TCGA) is the largest cancer genetic information database. Cervical cancer transcriptome profiling data and prognostic data were obtained from the TCGA consortium. The characteristics of the data are as follows: disease type (cervical squamous cell carcinoma), data category (Transcriptome Profiling), data type (Gene Expression Quantification), experimental strategy (RNA-Seq) and workflow type (HTSeq - Counts). The other filters were kept as default. Finally, data from a cohort containing 3 normal cervical squamous samples and 252 cervical squamous carcinoma samples were obtained from TCGA. Oncomine (version 4.5) (www.oncomine.org) is an open database containing 715 datasets and 86,733 samples. Three datasets in Oncomine: the Scotto Cervix (3 cervix squamous epithelium and 32 cervical squamous cell carcinoma), Pyeon Multi-cancer (8 cervix uteri and 20 cervical cancer), Biewenga Cervix (5 cervix uteri and 40 cervical cancer) datasets were chosen to validate the results obtained from TCGA (Table S1).

### Identification of differentially expressed genes (DEGs)

The transcriptome profiling data files for analysis were systemized and transferred into a txt file, which included expression and prognosis data using a Perl order line. Then, the package “edgeR” of Bioconductor (version 3.4) was applied in RStudio (version 3.3.2) to screen out the DEGs with a fold change >2, a p-value < 0.05 and a FDR value < 0.05, which were considered statistically significant^10^. Hierarchical clustering analysis was applied to categorize the data into two groups with similar expression patterns between cervical cancer and normal cervical epithelia. Expression data were obtained from the expression matrix, and for homogeneity, all expression values were log_10_ transformed. Each line in the heatmap corresponds to the expression of one gene in different samples.

### Kyoto Encyclopedia of Genes and Genomes (KEGG) pathway enrichment analysis of DEGs

As a knowledge base for the systematic and comprehensive analysis of gene functions in pathways, the KEGG links genomic information with higher-level function information. The Database for Annotation, Visualization and Integrated Discovery (DAVID) database (http://david.ncifcrf.gov, version 6.8) is an important foundation for high-throughput gene functional analysis. ClueGO (version 2.2.3) is a plug-in app of Cytoscape (http://www.cytoscape.org/) that allows for KEGG pathway enrichment analysis using databases other than the DAVID database. To analyze the DEGs at the functional level, KEGG pathway analysis was performed using both the DAVID online tool and ClueGo. Pathways including 8 or more DEGs are shown in the ClueGo-KEGG figures. A p-value < 0.05 was considered statistically significant.

### Pathway gene signatures analyzed using Gene Set Enrichment Analysis (GSEA)

GSEA is a computational method for exploring whether a given gene set is significantly enriched in a group of gene markers ranked by their relevance with a phenotype of interest^11^. The curated KEGG pathway V6.0 dataset was used to compare the impaired pathways in cervical cancer with normal samples. Additionally, the gene sets containing less than 15 genes or more than 500 genes were excluded. The phenotype label was set as cancer vs. normal. The t-statistic mean of the genes was computed in each KEGG pathway using a permutation test with 1,000 replications. The up-regulated pathways were defined by a normalized enrichment score (NES) >0, and the down-regulated pathways were defined by an NES < 0. Pathways with an FDR-P-value ≤ 0.1 were considered significantly enriched.

### Integration of protein-protein interaction (PPI) network and module analysis

The Search Tool for the Retrieval of Interacting Genes (STRING) database is an online tool designed to evaluate protein-PPI information. The STRING database (version 9.0) (http://string91.embl.de) includes 5,214,234 proteins from 1,133 organisms. To observe the interactive relationships among DEGs, we mapped the DEGs to STRING and selected the experimentally validated interactions with a combined score >0.4. Then, PPI networks were mapped using the Cytoscape software (version 3.40) (www.cytoscape.org). The plug-in app Molecular Complex Detection (MCODE) was used to screen the modules of the PPI networks in Cytoscape. The criteria were set as follows: MCODE scores >10 and number of nodes >10. Moreover, function and pathway enrichment analyses were performed for DEGs in the modules. A p-value < 0.05 was considered to be a statistically significant difference.

### Survival analysis of distinct gene sets and genes in cervical cancer

Single-sample GSEA (ssGSEA), an extension of GSEA, calculates separate enrichment scores for each sample and gene set pair. A ssGSEA score based on the expression of genes within each KEGG pathway was calculated for all cervical cancer samples in TCGA cohort. Cancers with a ssGSEA score above the cohort median were considered to have high expression levels of a particular KEGG pathway gene set, and cancers with a ssGSEA score below the cohort median were considered to have low expression levels of the KEGG pathway gene set. For each KEGG pathway, a Kaplan-Meier curve was constructed to compare the overall survival of cervical cancer patients with high expression of the KEGG pathway gene set against those with low expression levels of the KEGG pathway gene sets. A log-rank test was used to calculate the statistical significance of the difference in survival between the two groups. A Cox univariate hazard ratio was calculated as a measure of the magnitude of the difference in survival between the two groups^11,12^. Survival analyses of single genes in survival-related gene sets were conducted to evaluate the clinical significance of each gene. The “Survival” package was applied in RStudio, and Kaplan-Meier curves were mapped based on the follow-up data from TCGA. A log-rank test was used to calculate the statistical significance of the difference in survival between the two groups, a p-value < 0.05 indicates a statistically significant difference.

### Immunofluorescence

A total of 1 × 10^5^ cells were plated on coverslips overnight and fixed in 10% formalin following 5 µm camptothecin treatment for 2 hours. Detection of γH2A.X was carried out. Briefly, following cell permeabilization (0.25% TritonX-100, 15 minutes) and blocking (2% BSA, 30 minutes), a 1/250 dilution of Alexa Fluor® 647 conjugated rabbit monoclonal anti-PhosphoHistone-H2A.X (Ser139) (9720, Cell Signaling) was incubated with the cells for 1 hour. A 1/10,000 dilution of Hoechst 33342 (H3570, Invitrogen) was incubated with the cells for 5 min to stain the nuclei. Coverslips were mounted on the slides using Mowiol® 4–88 (81381, Sigma-Aldrich). Confocal imaging was performed using an Olympus FV1200 confocal and a 20X and 40X objective. Data are from two independent experiments with two coverslips per condition per experiment. Overall, at least 600 cells were counted per condition per independent experiment; additional details are described by a previous study^13^. Image analyses were performed using ImageJ v1.49.

### Quantitative real-time PCR analysis

Total RNA was extracted from cell lines with RLT reagent and 1% β-mercaptoethanol in the RNeasy Mini Kit (Qiagen, Cat. Number: 74106), and cDNA was produced using the High-Capacity cDNA Reverse Transcription Kit (Applied Biosystems, Cat. Number: 4368814). PCR was performed on an Applied Biosystems 7700 Prism real-time PCR machine with the QPCR Mix SYBR green and specific gene primers. Expression analysis by real-time PCR was done with the following primers: HIST1H2BD-F1 GATGCCTGAACCTACCAAGT and HIST1H2BD-R1 GCTTCTTCCCGTCCTTCTTC; HIST1H2BH-F1 ATGCCTGATCCAGCTAAGTC and HIST1H2BH-R1 CGTTTACGCTTCTTGCCATC; HIST1H2BJ-F1 CTGACACCGGCATTTCGTC and HIST1H2BJ-R1 CGGCCTTAGTACCCTCGGA; HIST1H2AM-F1 GGTGTTCTGCCTAACATCCA and HIST1H2AM-R1 CAGCCCTTACTTGCCCTTAG; and HIST1H4K-F1 AAGGCGGGAAGGGTCTT and HIST1H4K-R1 CTTGGTGATGCCCTGGATATTG. The expression of target genes was normalized to the expression of human 18 S ribosomal RNA. For fold change analysis, the data were transformed using the ‘relative standard curve and comparative threshold cycle (Ct) method (∆∆Ct)’ as described by Applied Biosystems and in previous studies. The results of the other cell lines were normalized according to the results of ME180. Results are expressed as relative gene expression for each target gene^14,15^.

### Statistical analysis

Statistical analyses were performed using GraphPad Prism software (version 7.0c), and data were displayed as the mean and standard error of measurement (SEM). The difference between two groups was analyzed using an unpaired Student t-test, and P < 0.05 was considered statistically significant^16^.

## Results

### Workflow for the identification of key pathways and genes in cervical cancer

**Figure 1:**
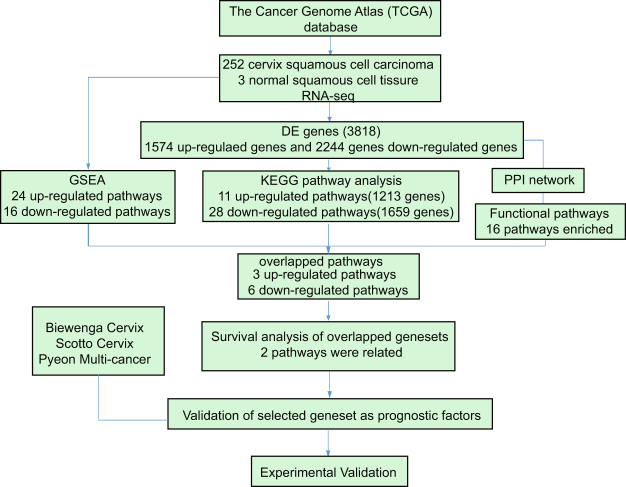
Workflow. Workflow for the identification of key pathways and genes between cervical cancer and normal samples.

We aimed to identify the differentially expressed genes (DEGs) between tumor and normal tissues and find significant signatures for cervical cancer. After being obtained based on the TCGA cervical cancer RNA-Seq database, DEGs were mapped for KEGG pathway and Protein-protein interaction (PPI) network analyses. KEGG pathway enrichment analyses were conducted for genes in each PPI module and a list of pathways in every module were obtained. Gene sets enrichment (GSEA), KEGG and the PPI modules (PPI-m) pathways were merged to screen out the most significant pathways between cervical cancer and normal tissues. Then, we performed survival analysis on the overlapped pathways. The DNA replication and systemic lupus erythematosus (SLE) pathways were selected as distinct signatures by the different enrichment methods and the genes included in these two pathways were highly correlated with survival. SLE is a multifactorial autoimmune disease that results from a combination of genetic, epigenetic and environmental factors^17^. Histones were first implicated to in SLE pathogenesis in 1979 with the description of specific antibodies^18^. Antibodies against a subset of nucleosomes which contain the protein HMG-17 were also described in SLE^19^. With further research, anti-histone antibodies^20^ and modifications of histones such as histone acetylation/deacetylation and methylation/demethylation were determined as one of the important contributors to the pathogenesis of SLE^21,22^. Here we identified 16 histone family genes previously enriched in the SLE pathway, which are associated with survival of cervical cancer patients. These histone family genes play a role in DNA repair. In addition, the majority of these histones encoding genes were overexpressed in 2 out of 4 cervical cancer cell lines, which prompted the evaluation of the DNA repair capacity of these 4 cell lines. Overlapping the 16 genes with the Oncomine datasets, led to the selection of a subset of five genes which overexpressed in cancer cervical tissue and crossed three independent studies. Finally, experimental validation and survival analyses of these five genes were conducted. The results from multiple datasets show that our proposed approach not only improves survival prediction but also identifies significant biological pathways (Fig. 1).

### Identification of differentiate expression genes (DEGs)

**Figure 2:**
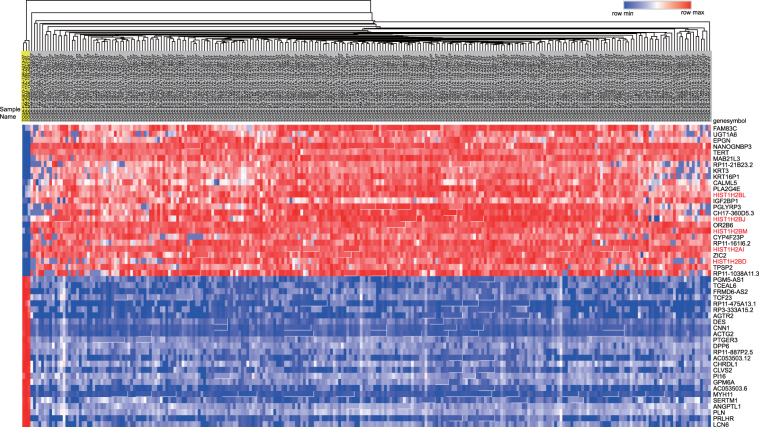
Heatmap of the top 25 up- and down-regulated DEGs. The top 25 up- and down-regulated DEGs are shown in the heatmap. The red color represents high expression, and the blue color represents low expression. Normal samples are indicated by a yellow background.

To investigate important gene signatures for cervical cancer, we performed a DEG analysis between 3 normal samples and 252 cervical squamous cancer samples by the R package “edgeR”. Ultimately, a total of 3,818 genes were identified, of which 1,574 were up-regulated and 2,244 were down-regulated. A heatmap (top 25 up-regulated and 25 down-regulated genes) is shown in Fig. 2. The red color represents high expression, including histone variant genes (HIST1H family genes), and the blue color represents low expression. The gene list is provided in Table S2.

### Identification of pathways based on DAVID and ClueGO

**Figure 3:**
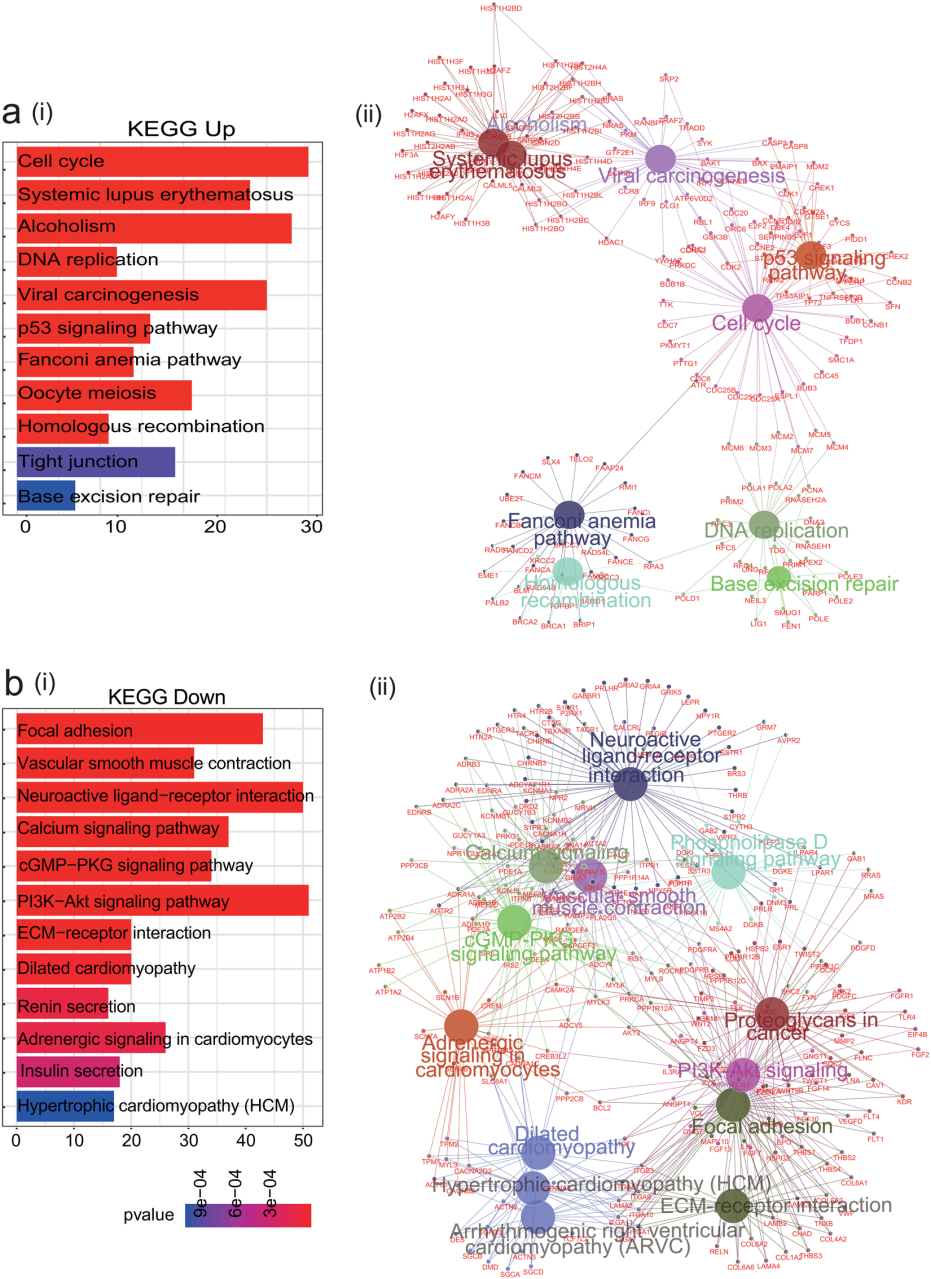
Identification of pathways based on DAVID and ClueGO. (**a**) The most significant pathways enriched in the up-regulated DEGs were related to cell cycle, systemic lupus erythematosus, alcoholism, etc. based on the DAVID database (i), whereas the ClueGO result showed that cell cycle, systemic lupus erythematosus, DNA replication, etc. were significantly enriched (ii). (**b**) Down-regulated pathways, namely those related to neuroactive ligand-receptor interactions, calcium signaling, ECM-receptor interactions, etc., were identified using the same method (i,ii).

KEGG pathway is a collection of pathway maps representing known molecular interactions and reaction networks for metabolism, genetic information processing, environmental information processing, cellular processes, organismal systems, human diseases and drug development. To further explore the function of DEGs previously identified, KEGG pathway analyses were conducted. Significantly enriched pathways between tumor and normal tissues, with *p*-value < 0.05 were identified with DAVID (gene functional classification) and ClueGO (gene ontology integration), the two most frequently-used tools to carry out KEGG pathway enrichment analysis (Table S3, 4). Based on the DAVID database, some of the most significantly enriched pathways in the up-regulated DEGs were cell cycle, SLE, and alcoholism (Fig. 3ai), whereas ClueGO showed that cell cycle, SLE, DNA replication, were significantly enriched (Fig. 3aii). Importantly, the 5 pathways enriched in ClueGO, including the SLE pathway, were validated on the DAVID database. Interestingly, the SLE pathway mainly consists of histone family genes (histone cluster 1 H2A and H4 family). Using the same method, we determined the down-regulated pathways, namely those involved in neuroactive ligand-receptor interactions, calcium signaling, ECM-receptor interactions, etc. (Fig. 3bi,ii).

### Identification of key gene signatures in cervical cancer by KEGG pathway, GSEA and Protein-protein interaction analysis

**Figure 4:**
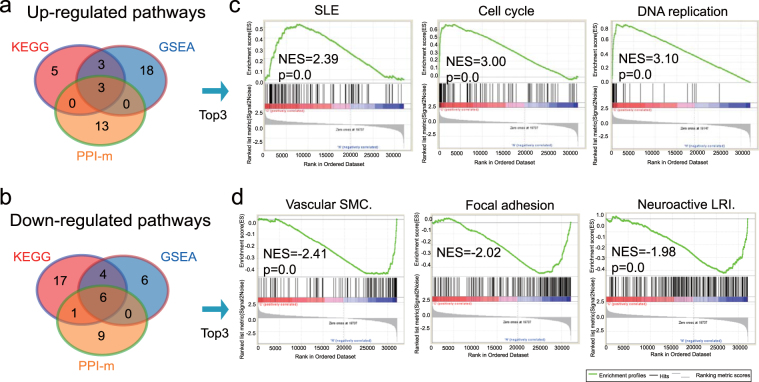
Overlap of up- and down-regulated pathways among KEGG, GSEA and PPI. (**a**) A total of 3 up-regulated pathways were overlapped in the KEGG, GSEA and PPI: the SLE, cell cycle and DNA replication pathways. (**b**) A total of 7 down-regulated pathways were overlapped between KEGG, GSEA and/or PPI, including pathways involved in calcium signaling, focal adhesion, cell adhesion molecular, etc. (**c**) The GSEA results of the three pathways are shown, demonstrating that the DNA replication, cell cycle and SLE pathways were significantly increased, with an NES of 3.10, 3.00 and 2.39, respectively, in cervical squamous cell cancer patients. (**d**) The GSEA results of the top three pathways are shown, demonstrating that the vascular smooth muscle contraction, focal adhesion, and neuroactive ligand-receptor interaction pathways are significantly decreased, with an NES of −2.41, −2.02, −1.98, respectively, in cervical squamous cell cancer patients.

GSEA is a computational method for exploring whether a given gene set is significantly enriched in a group of gene markers ranked by their relevance with a phenotype of interest. As another new method for pathway analysis, GSEA is focused on the whole gene set expression other than DEGs between cancer and normal tissue. The curated KEGG pathway V6.0 dataset was used to compare the impaired pathways in cervical cancer compared to normal samples. Based on the same RNA-Seq data we used previously, GSEA analysis showed that a total of 24 pathways were up-regulated in the cancer group, while 16 pathways were up-regulated to compare with the normal group (Tables S5 and S6). Protein-protein interaction (PPI) analysis was performed to understand the system-level of functional interactions of these DEGs based on information in the STRING database. After mapping DEGs into STRING, a total of 3,818 nodes and 17,128 edges were analyzed using plug-in MCODE. KEGG pathway enrichment analyses were subsequently conducted for genes in each PPI-m and a list of pathways in every module was obtained. These pathways were mainly associated with Neuroactive ligand-receptor interaction, Calcium signaling pathway, Chemokine signaling pathway, ECM-receptor interaction, SLE, DNA replication and also Cell cycle (Table S7). To further understand these pathways, we looked to identify overlapping pathways between those identified by the three methods used (i.e. KEGG, GSEA and PPI) (Fig. 4a,b); three overlapping up-regulated pathways were identified: DNA replication, cell cycle and SLE (Fig. 4c), which were all related to DNA repair, replication and the immune system, whereas six mutual down-regulated pathways were identified: vascular smooth muscle contraction, focal adhesion, neuroactive ligand-receptor interaction, calcium signaling, dilated cardiomyopathy and ECM-receptor interaction pathways. The GSEA enrichment results of top 3 (ranked by *p* value and FDR *q* value) down-regulated pathways are shown in Fig. 4d. Positive normalized enrichment score (NES) values in SLE, cell cycle and DNA replication (2.39, 3.00, 3.10, respectively) and upper-left peaks in these three GSEA curves mean that most of the genes in these pathways are up-regulated; conversely, negative NES values in vascular smooth muscle contraction, focal adhesion, neuroactive ligand-receptor interaction, calcium signaling, dilated cardiomyopathy and ECM-receptor interaction pathways (−2.41, −2.02, −1.98, −1.92, −1.82, −1.64, respectively) and lower-right peaks in these GSEA curves mean that most of the genes in these pathways are down-regulated.

### Gene set of histone family predicts better prognosis

**Figure 5:**
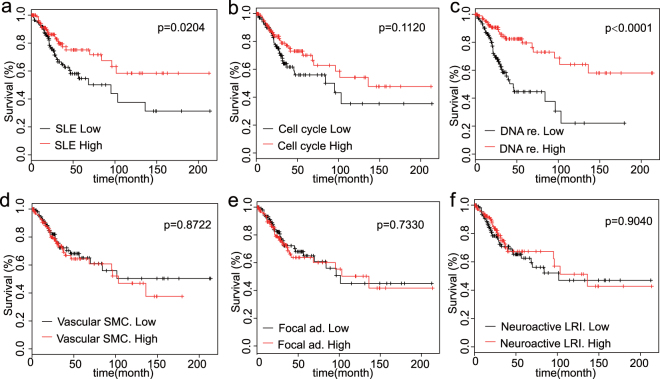
Gene set survival curves of different pathways. (**a**–**c**) The overlapped pathways in KEGG, GSEA and PPI-m were analyzed to explore their relationship with survival. In the up-regulated pathways, SLE and DNA replication were significantly related to the survival rate, with p equaling 0.0204 and 0.0004, respectively, whereas cell cycle was not correlated with the survival rate. (**d**–**f**) In the down-regulated pathways, the vascular smooth muscle contraction, focal adhesion and neuroactive ligand-receptor interaction pathways were not correlated with the survival rate.

To further investigate the genes in overlapped significant pathways, we obtained mutual genes in different KEGG pathways based on both DEGs and PPI modules. (Tables S3, S4 and S7). Gene set survival analysis was performed on these genes. The results demonstrated that the overall survival of patients with numerous DEGs (HIST1H3J, HIST1H2BD, HIST1H4K, HIST1H2AD, HIST1H2BH, HIST1H2BO, HIST1H2BL, HIST1H2BJ, HIST1H4E, HIST1H3C, HIST1H3D, HIST1H2AJ, HIST1H2AM, HIST3H2BB, HIST1H3F and HIST1H2AL) in the SLE pathway was significantly improved compared to those with fewer DEGs in the SLE pathway (p = 0.02104). The DNA replication signature was also related to survival (p < 0.0001) (Fig. 5a,b), whereas the other pathways were not significantly associated with survival (Fig. 5c–f).

### Verification of differentially expressed histone signatures

**Figure 6:**
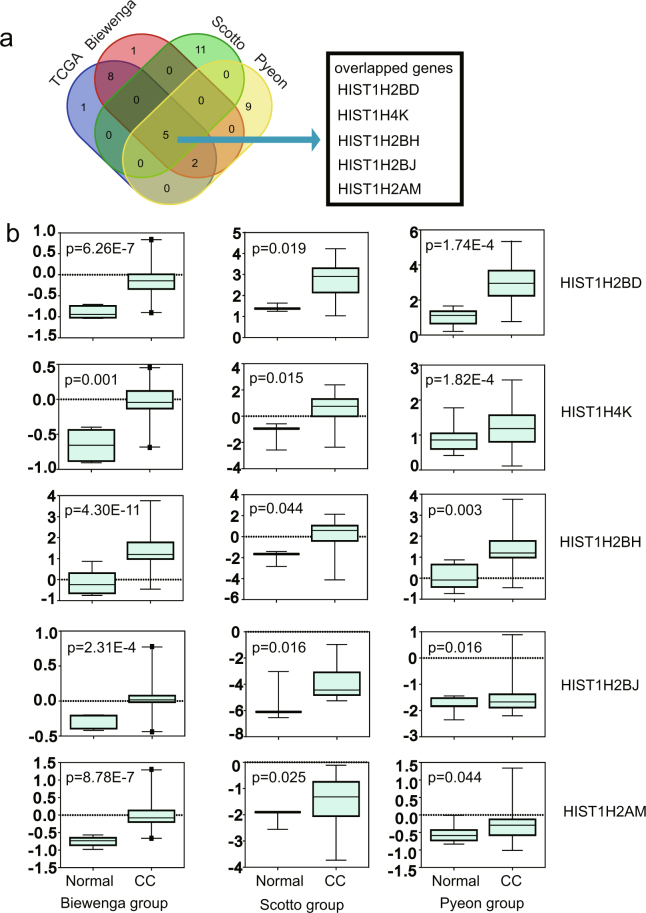
Box plots of histone genes based on Oncomine datasets. (**a**) Five histone genes, HIST1H2BD, HIST1H2BH, HIST1H2BJ, HIST1H2AM and HIST1H4K, were identified after using the Oncomine database to verify the histone gene set obtained using TCGA database. (**b**) All five genes were significantly overexpressed in the Scotto Cervix, Pyeon Multi-cancer and Biewenga Cervix datasets.

Scotto Cervix (3 cervix uteri and 32 cervical cancer), Pyeon Multi-cancer (8 cervix uteri and 20 cervical cancer) and Biewenga Cervix (5 cervix uteri and 40 cervical cancer) datasets obtained from the Oncomine database were used to validate the aberrant expression of histone genes in cervical cancer. The Biewenga Cervix dataset revealed that 16 histone genes were up-regulated in the cancer group. A total of 7 out of 16 genes were confirmed in the Pyeon Multi-cancer cohort and 5 (HIST1H2BD, HIST1H4K, HIST1H2BH, HIST1H2BJ and HIST1H2AM) out of these 7 genes were also verified in the Scotto Cervix cohort (Fig. 6a). The expression of the 5 histone genes commonly identified in three datasets from Oncomine is shown in Fig. 6b.

### Expression of histone variants and its relationship with DNA repair in cervical cancer cells

**Figure 7:**
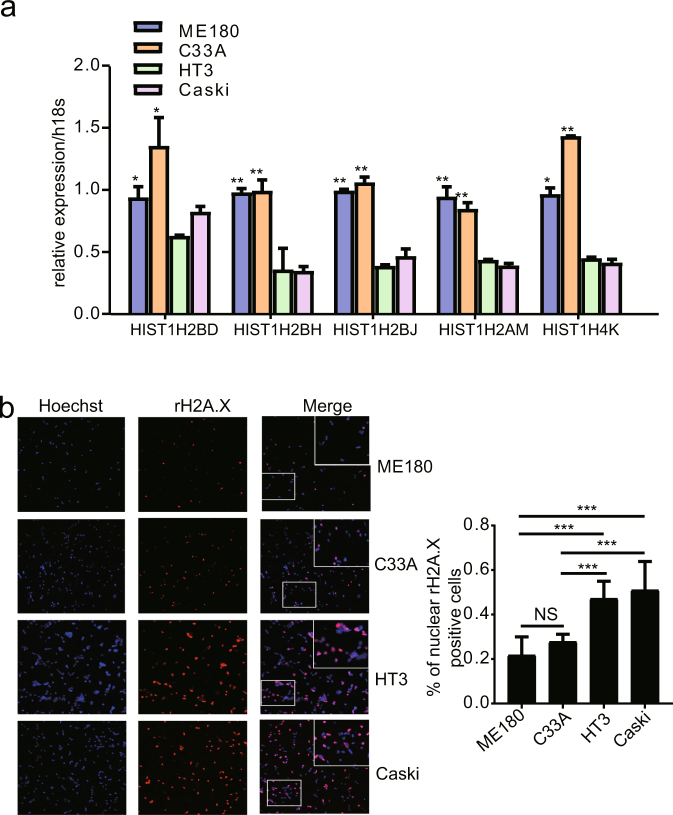
Functional experiments regarding the histone signature. (**a**) The expression levels of the 5 histone genes were determined in 4 HCC cell lines (ME180, C33A, HT3 and Caski). The histone gene expression level in the ME180 and C33A cell lines was significantly higher than that in the HT3 and Caski cell lines. (**b**) Micrographs showing the DNA damage marker γH2A.X in the cervical cancer cell lines mentioned above after treatment with 5 µM camptothecin for 2 hours. Non-treated images are not shown here. Cell nuclei are stained blue by Hoechst dye to indicate the cell number. The graph to the right of the micrographs shows the quantification of the proportion of cells with γH2A.X-positive nuclei among 600 cells from 6 images acquired using a 20X objective. The data were analyzed using the Analyze Particles tool in ImageJ; points larger than 30 pixels 2 were counted as a cell.

To further investigate the biological functions of the 5 histone variant genes identified above (HIST1H2BD, HIST1H4K, HIST1H2BH, HIST1H2BJ and HIST1H2AM), 4 HCC cell lines (ME180, C33A, HT3 and Caski) were used. The mRNA expression levels of these 5 histone genes were significantly higher in the ME180 and C33A cell lines compared with the HT3 and Caski cell lines (Fig. 7a). The histone variants represent key chromatin components that facilitate DNA repair, indicating that ME18- and C33A cells should exhibit less DNA damage upon genotoxic challenge. In line with this, γH2A.X immunofluorescence of the 4 cervical cancer cell lines after camptothecin-induced DNA damage showed decreased nuclear staining in ME18- and C33A cells compared to HT3 and Caski cells (Fig. 7b). Given that γH2A.X levels are directly related to the amount of DNA damage present, these results suggest that ME180 and C33A cells are less prone to DNA damage that the other cell lines, correlating with a protective role of increased histone expression.

### Histone gene sets may be independent prognostic factors for cervical squamous cell cancer patients

**Figure 8:**
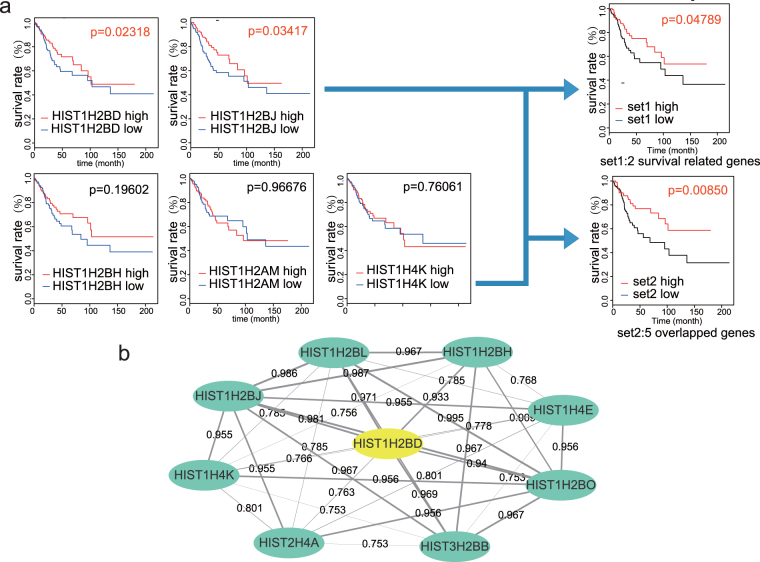
Survival curves of 5 overlapped genes between the experimentally validated genes and the Oncomine data. (**a**) Kaplan-Meier survival curves of HIST1H2BD, HIST1H4K, HIST1H2BH, HIST1H2BJ and HIST1H2AM were used to explore the relationship between the expression of genes and the survival rate. The expression of HIST1H2BD and HIST1H2BJ was significantly correlated with the survival rate of cervical squamous cell cancer patients, with a p-value equaling 0.02318 and 0.03417, respectively. The expression of the other 3 genes was not significantly correlated with the survival rate. Survival analyses of two gene sets (HIST1H2BD and HIST1H2BJ; and HIST1H2BD, HIST1H4K, HIST1H2BH, HIST1H2BJ and HIST1H2AM) demonstrated that both gene sets were related to survival (p = 0.04789 and 0.00850, respectively). (**b**) Protein-PPI network analysis of histone genes enriched in the SLE pathway. HIST1H2BD, which acted as a seed gene, is marked in yellow. Nodes denote proteins, and edges denote interactions. The width of each line represents the combined-score of interaction between two nodes (the score is also marked on each edge).

To further explore the clinical data pertaining to the 5 histone genes (HIST1H2BD, HIST1H4K, HIST1H2BH, HIST1H2BJ and HIST1H2AM), we analysed survival using the “Survival” package in R based on TCGA datasets. The expression levels of HIST1H2BD and HIST1H2BJ were significantly related to the overall survival of cervical squamous cell cancer patients (p = 0.02318 and 0.03417, respectively), whereas the expression levels of the other 3 genes did not. As such, high expression of HIST1H2BD and HIST1H2BJ was associated with prolonged patient survival. High expression of the following two gene sets was also significantly correlated with the prognosis of cervical cancer patients: HIST1H2BD and HIST1H2BJ; and HIST1H2BD, HIST1H2BJ, HIST1H2BH, HIST1H2AM and HIST1H4K (p = 0.04789 and 0.00850, respectively, Fig. 8a). PPI network analysis is a powerful tool for understanding biological responses in health and disease. The DEGs related to DNA repair (SLE)-mediated signaling, including histone family genes, were mapped onto the reference network obtained from the STRING database. The resulting network was formed into subnetworks, each of which represented protein subcomplexes or functional modules. Subsets of histone family genes were expressed together, functioned together and may serve as a prognosis maker for cervical cancer, which further supported our hypothesis. HIST1H2BD, which acted as a seed gene in this submodule, is marked in yellow (Figs 8b and S1).

## Discussion

Cervical cancer is associated with high recurrence and mortality rates due to a lack of knowledge in the gene signatures related to its pathogenic development. Thus, identifying key molecular signatures associated with cervical cancer progression will identify useful predictive markers. Here, an integrative strategy was utilized to systematically analyze the aberrant expression patterns of cervical cancer based on RNA-Seq data, gene profiling and molecular signature analysis. In the present study, we extracted data from TCGA and identified 1,574 up-regulated and 2,244 down-regulated DEGs between cervical cancer patients and normal controls using bioinformatics analysis. Functional annotation showed that these DEGs in 11 up-regulated and 28 down-regulated pathways were mainly involved in cell cycle, DNA replication, chemical homeostasis, and immune response, etc. Furthermore, 16 enriched genes were identified from the protein-PPI network, and subnetwork analyses revealed the genes involved in each significant pathway. Then, we combined the cross-validated prognostic scores of all pathways that were obtained in the first step as new predictors to build an integrated prognostic model for prediction. PPI analysis of the SLE pathway revealed 9 functional interaction modules of histone variants (HIST1H2BO, HIST1H4A, HIST1H2BD, HIST1H2BL, HIST1H2BJ, HIST1H2BH, HIST1H4E, HIST1H4K and HIST3H2BB) that are associated with the prognosis of cervical cancer patients (Fig. 8). These network modules are related to DNA repair, and SLE/histone variant-mediated signaling pathways. In our validation study, the expression of histone variants was significantly associated with clinical factors, including a better prognosis, and negatively associated with the expression of the DNA damage marker γH2AX in the nucleus after DNA damage, which suggests a correlation with the DNA repair mechanism. Two sets of histone variant genes were significantly associated with the overall survival of patients with cervical cancer in TCGA cohorts.

Identification of robust prognostic gene signatures as biomarkers using genomics data can be challenging. We have developed a more efficient method for the identification of prognostic biomarkers in cancer gene expression datasets using modules derived from a highly reliable gene functional interaction network. When applied to cervical cancer, we discovered a novel 7-gene signature associated with patient survival. The signature was replicated across 3 independent gene expression studies, and this approach was similar to other recent studies^23–25^. We propose that this strategy allows for the simple and rapid analysis of cancer-dysregulated gene expression data to identify novel prognostic network modules.

We compared the expression of histone variant genes, including the H2B family subset, and other genes that are overexpressed in cervical squamous cell cancer patients with normal subjects from TCGA. We also investigated three different datasets from Oncomine. Surprisingly, there was a wide range of expression levels among the individual genes, suggesting that the regulation of histone transcription is gene specific and more complex than we originally assumed; even the different cell lines exhibited distinct expression patterns of total histone variant genes, including the main subgroup H2B mRNA. The efficiency of transcription could be regulated via the promoter or the 3′ UTR sequence; the stem loop sequence can affect the transcriptional processing of the 3′ end, there are probably transcriptional and post-transcriptional regulations of the mRNA level/stability involved here (TFs and microRNAs)^26^. Most interestingly, 6 out of 11 highly expressed H2B subgroup genes were co-expressed together in the same cell context, which supported protein-protein interaction modules of histone variants and may be sufficient to allow for DNA repair functions.

We were not able to assess the protein expression levels of each specific variant because specific antibodies were not commercially not available. There are 4 H2A and 6 H2B histone variants; in our case, the module contains 9 genes in the same pathway or protein-PPI module, thus allowing us to shift away from the previous methods of focusing on single genes as biomarkers. The protein-PPI network approach relies on the strength of the gene-to-gene interactions, which are closely related to their function. The entire SLE histone variant module, rather than one of its components, may be considered a predictive biomarker (Fig. 8).

Our study revealed that one of those mechanisms involves the up-regulation of histone variant genes and several pathways, including the SLE pathway. According to the KEGG pathway analysis, the H2A, H2B, H3 and H4 histones genes involved in the function of autoantigens are related to the SLE disease due to active MHCII, B cell receptor signaling and autoantibodies. In general, H2A and H2B histones form octamers with H3 and H4 histones and are involved in the packaging of DNA into nucleosomes. Thus, the evaluated histone transcript levels may be indicative of a stalled cell cycle as cells struggle to repair the chemotherapy-induced DNA damage. Consistent with that interpretation, our data supported that low expression of histone variants in cervical cancer cell lines was associated with a significantly higher amount of DNA damage after DNA damage, which suggests a correlation with the DNA repair mechanism. Current studies have shown increased levels of DNA double-strand breaks (DSBs) in the tumor cells of clinical specimens from various tissues or tumor cell cultures. Some studies have reported the possible utility of γH2AX measurements in clinical diagnostics, including the differential diagnosis of metastatic renal cell carcinoma or the monitoring of ulcerative colitis, which predisposes patients to colorectal cancer; chronic inflammatory disease have been associated with shorter telomeres, which have been associated with chromosomal instability and tumor progression, and therefore, γH2AX may be used to screen for patients with genomic instability. Measurements of γH2AX may be a valuable way to detect other, perhaps undiscovered, conditions that affect DNA repair and may predispose patients to cancer. In addition, increased numbers of γH2AX expression foci are significantly associated with shorter survival in breast carcinoma^27^, endometrial carcinoma^28^, and non-small cell lung cancer patients^29^. In prostatic cancer, higher expression of γH2AX is associated with chemoresistance due to the induction of G2/M arrest in cancer stem-like cells^30^. Thus, higher expression of γH2AX might be useful for the prediction of survival in soft-tissue sarcoma (STS) patients and may be a potential therapeutic target for STS patients^31^. Those results support our finding that high expression of a subset of histone variant genes with low expression of γH2AX suggests a better outcome in cervical cancer patients.

H3 and H4 histone modification patterns strongly associate with either an active or a repressed gene transcription status. The current understanding of H2A and H2B histone modification is based on studies in yeast and a few tumor cell lines; nonetheless, a few important features have been revealed regarding the modified sites of acetylation, phosphorylation and ubiquitination. Since data-mining from TCGA database revealed that the expression of the histone H2B K monoubiquitin (H2Bub1) ligase RNF20 is dysregulated in cancer, we elucidated its effect on transcription and cancer progression. H2B forms a (H2A-H2B)-2 tetramer. This tetramer and its component dimers are easily exchanged in and out of the nucleosome compared to H3 and H4, indicating that the modifications on H2A and H2B are less likely to be maintained in chromatin^32^. For this reason, modifications on H2A/H2B have been less studied than those on H3 and H4 in the epigenetics field. However, many studies acknowledge the potential impact that modifications on H2B can have on chromatin dynamics^33^. Some forms of epigenetic modifications alter the chromatin structure to alter gene expression, which affects pathological processes in a multitude of disease conditions related to tumorigenesis. Regulators of DNA-associated signaling networks play critical roles that are required for physiological processes such as cell growth, cycling, movement, and DNA repair - all essential features of human cancer^34,35^.

Based on gene expression profiling data from multiple cohorts, our findings demonstrate that increased histone variant expression is a strong prognostic biomarker in cervical cancer patients. Histone variant expression measurements may help to select cervical cancer patients who may benefit from radiation therapy. Gene functional interaction modules that are likely associated with cervical cancer patient prognosis were also identified. These network modules were related to DNA repair-mediated signaling pathways. Taken together, our results provide the first evidence that two SLE (histone variant) gene sets (HIST1H2BD and HIST1H2BJ; and HIST1H2BD, HIST1H2BJ, HIST1H2BH, HIST1H2AM and HIST1H4K) might be independent prognostic factors for better survival in cervical cancer patients.

## Electronic supplementary material

supplementary information
