# Supplementary material for: Identification of a histone family gene signature for predicting the prognosis of cervical cancer patients
Source: Sci Rep. 2017 Nov 28;7:16495. doi: 10.1038/s41598-017-16472-5 (PMC5705706; doi:10.1038/s41598-017-16472-5)
Supplement: Supplementary file 1 — supplementary information [file 41598_2017_16472_MOESM1_ESM.pdf]

Supplementary Material

Identification of a histone family gene signature for predicting the prognosis of cervical cancer patients

Xiaofang Li[1#], Run Tian[2#], Hugh Gao[3], Yongkang Yang[1], Bryan RG Williams[3], Michael P. Gantier[3], Nigel A.J. McMillan[4], Dakang Xu[3,5], Yiqun Hu[5]* and Yan'e Gao[1]*

[1]Department of Obstetrics and Gynecology, the Second Affiliated Hospital, Xi'an Jiaotong University, Xi'an, P. R. China

Corresponding Author: Tel. +86 29 87679558; E-mail: drgao1991@hotmail.com

Table S1 Information of the data resources

Groups	Cervix Uteri (number of samples)	Cervical Carcinoma (number of samples)	Experiment type
TCGA (TCGA-CESC) #	3	252	RNA-sequencing
Biewenga Cervix (GSE7410)[1]*	5	40	Micro array(mRNA)
Pyeon Multi-cancer (GSE6791)[2]*	8	20	Micro array(mRNA)
Scotto Cervix (GSE9750)[3]*	3	32	Micro array(mRNA)

Abbreviations: TCGA: The Cancer Genome Atlas

#TCGA-CESC: project ID of the data we used from TCGA

*The three datasets we used for validation also can be searched in GEO(Gene Expression Omnibus microarray data repository), GEO accessions and references were shown.

References:

1 Biewenga, P. *et al.* Gene expression in early stage cervical cancer. *Gynecologic Oncology* **108**, 520-526, doi:10.1016/j.ygyno.2007.11.024.

2 Pyeon, D. *et al.* Fundamental differences in cell cycle deregulation in human papillomavirus-positive and human papillomavirus-negative head/neck and cervical cancers. *Cancer research* **67**, 4605-4619, doi:10.1158/0008-5472.CAN-06-3619 (2007).

3 Scotto, L. *et al.* Identification of copy number gain and overexpressed genes on chromosome arm 20q by an integrative genomic approach in cervical cancer: Potential role in progression. *Genes, Chromosomes and Cancer* **47**, 755-765, doi:10.1002/gcc.20577 (2008).

Table S2 Information of the DEGs corresponding to the heatmap (Figure 2)

	logFC	logCPM	P-Value	FDR
**Up-DEGs**				
**(cancer versus normal)**				
FAM83C	11.00726	4.921006	0.001449	0.012618
UGT1A6	10.5887	1.75696	0.007979	0.048456
EPGN	10.43008	1.599564	0.008235	0.04946
NANOGNBP3	10.208962	1.85087	0.000772	0.007596
TERT	10.04358	1.217666	3.68E-05	0.000559
MAB21L3	9.786823	0.9653	0.000204	0.002506
RP11-21B23.2	9.699783	0.87928	0.008012	0.048596
KRT3	9.606661	0.788798	0.008165	0.049208
KRT16P1	9.605554	0.786461	0.00532	0.035572
CALML5	9.491751	7.428736	0.00544	0.036201
PLA2G4E	9.480202	4.407806	0.003248	0.024052
HIST1H2BL	9.372446	3.376436	0.006084	0.039312
IGF2BP1	9.302071	3.031891	0.007071	0.044205
PGLYRP3	9.283234	3.376436	0.006084	0.039312
CH17-360D5.3	9.093475	2.292376	0.000504	0.005383
HIST1H2BJ	9.020184	3.250386	0.001613	0.013761
OR2B6	9.008599	0.204147	3.27E-05	0.000503
HIST1H2BM	9.004763	2.004825	2.64E-05	0.000418
CYP4F23P	8.943169	0.140711	0.007992	0.048505
RP11-161I6.2	8.844568	0.045341	0.001989	0.01626
HIST1H2AI	8.832465	0.033357	1.11E-06	2.34E-05
ZIC2	8.736181	4.075299	3.05E-05	0.000474
HIST1H2BD	8.663077	4.532132	0.001339	0.011848
TPSP2	8.661043	-0.13167	0.005447	0.036211
RP11-1038A11.3	8.606365	-0.18447	0.00276	0.02112
**Down-DEGs**				
**(cancer versus normal)**				
PGM5-AS1	-9.16467	-1.34416	1.62E-88	5.21E-84
TCEAL6	-8.61211	-2.44778	7.55E-83	8.08E-79
FRMD6-AS2	-7.97443	-2.01225	3.94E-49	3.42E-46
TCF23	-7.95359	0.658118	9.46E-41	4.22E-38
RP11-475A13.1	-7.91355	-4.14075	3.47E-45	1.96E-42
RP3-333A15.2	-7.86555	-3.21927	8.93E-63	1.91E-59
AGTR2	-7.7095	-1.16863	5.24E-17	4.05E-15
DES	-7.58321	6.095272	1.18E-56	1.57E-53
CNN1	-7.52521	5.393158	7.45E-77	5.98E-73
ACTG2	-7.35287	5.862155	2.33E-56	3.00E-53
PTGER3	-7.2886	1.997712	2.51E-74	1.61E-70
DPP6	-7.21308	1.150937	7.32E-42	3.46E-39
RP11-887P2.5	-7.17872	-1.53145	1.71E-46	1.10E-43

AC053503.12	-7.12269	-3.22431	3.51E-27	7.38E-25
CHRDL1	-7.09942	2.029395	9.46E-34	2.87E-31
CLVS2	-7.08581	-2.73499	6.39E-28	1.40E-25
PI16	-7.07756	2.014868	4.14E-37	1.44E-34
GPM6A	-6.9051	-0.55645	4.90E-45	2.70E-42
AC053503.6	-6.90042	-3.74084	1.05E-23	1.61E-21
MYH11	-6.89444	6.838917	1.02E-56	1.48E-53
SERTM1	-6.84045	0.87733	9.81E-16	6.44E-14
ANGPTL1	-6.80391	0.742043	5.09E-50	4.96E-47
PLN	-6.71017	1.648954	8.63E-51	8.66E-48
PRLHR	-6.69165	-2.98097	3.67E-17	2.91E-15
LCN6	-6.67999	-2.36527	1.63E-45	9.52E-43

Table S3 Up-regulated KEGG pathways

ID	Description	Gene Ratio	P-value	Q-value	Gene ID
hsa04110	Cell cycle	35/368	0.0000	0.0000	BUB1/BUB1B/CCNA2/CCNB1/CCNB2/CCNE1/CCNE2/CDC20/CDC25A/CDC25C/CDC45/CDC6/CDC7/CDK1/CDKN2A/CDKN2B/CHEK1/E2F1/E2F2/ESPL1/MAD2L1/MCM2/MCM4/MCM5/ORC1/ORC6/PCNA/PKMYT1/PLK1/PTTG1/RBL1/SFN/SKP2/SMC1B/TTK
hsa05322	Systemic lupus erythematosus	28/368	0.0000	0.0000	HIST1H2AD/HIST1H2AG/HIST1H2AI/HIST1H2AJ/HIST1H2AL/HIST1H2AM/HIST1H2BC/HIST1H2BD/HIST1H2BF/HIST1H2BG/HIST1H2BH/HIST1H2BI/HIST1H2BJ/HIST1H2BL/HIST1H2BO/HIST1H3B/HIST1H3C/HIST1H3D/HIST1H3F/HIST1H3G/HIST1H3H/HIST1H3J/HIST1H4D/HIST1H4E/HIST2H2AB/HIST2H2BF/HIST2H4A/HIST3H2BB
hsa05034	Alcoholism	33/368	0.0000	0.0000	CALML3/CALML5/GNGT1/GRIN1/GRIN2D/HIST1H2AD/HIST1H2AG/HIST1H2AI/HIST1H2AJ/HIST1H2AL/HIST1H2AM/HIST1H2BC/HIST1H2BD/HIST1H2BF/HIST1H2BG/HIST1H2BH/HIST1H2BI/HIST1H2BJ/HIST1H2BL/HIST1H2BO/HIST1H3B/HIST1H3C/HIST1H3D/HIST1H3F/HIST1H3G/HIST1H3H/HIST1H3J/HIST1H4D/HIST1H4E/HIST2H2AB/HIST2H2BF/HIST2H4A/HIST3H2BB
hsa03030	DNA replication	12/368	0.0000	0.0000	DNA2/FEN1/LIG1/MCM2/MCM4/MCM5/PCNA/POLD1/POLE/POLE2/RFC4/RNASEH2A
hsa05203	Viral carcinogenesis	30/368	0.0000	0.0000	ATP6V0D2/BAK1/CCNA2/CCNE1/CCNE2/CCR8/CDC20/CDK1/CDKN2A/CDKN2B/CHEK1/HIST1H2BC/HIST1H2BD/HIST1H2BF/HIST1H2BG/HIST1H2BH/HIST1H2BI/HIST1H2BJ/HIST1H2BL/HIST1H2BO/HIST1H4D/HIST1H4E/HIST2H2BF/HIST2H4A/HIST3H2BB/IRF7/PMAIP1/RBL1/SKP2/SYK
hsa04115	p53 signaling	16/368	0.0000	0.0000	CCNB1/CCNB2/CCNE1/CCNE2/CD8

	pathway				2/CDK1/CDKN2A/CHEK1/GTSE1/PERP/PMAIP1/RRM2/SERPINB5/SFN/TP53AIP1/TP73
hsa03460	Fanconi anemia pathway	14/368	0.0000	0.0000	BLM/BRCA1/BRCA2/BRIP1/EME1/FAAP24/FANCA/FANCB/FANCD2/FANCI/RAD51/RMI1/RMI2/UBE2T
hsa04114	Oocyte meiosis	21/368	0.0000	0.0000	AURKA/BUB1/CALML3/CALML5/CCNB1/CCNB2/CCNE1/CCNE2/CDC20/CDC25C/CDK1/ESPL1/FBXO43/ITPR3/MAD2L1/PKMYT1/PLK1/PTTG1/RPS6KA1/SGOL1/SMC1B
hsa03440	Homologous recombination	11/368	0.0000	0.0001	BLM/BRCA1/BRCA2/BRIP1/EME1/POLD1/RAD51/RAD54B/RAD54L/TOPBP1/XRCC2
hsa04530	Tight junction	19/368	0.0010	0.0253	CGN/CLDN14/CLDN4/CLDN7/CRB3/EPB41L4B/F11R/LLGL2/MARVELD2/MARVELD3/MICALL2/MYH14/OCLN/PCNA/PRKCZ/RUNX1/SLC9A3R1/TUBA1C/TUBA4A
hsa03410	Base excision repair	7/368	0.0012	0.0258	FEN1/LIG1/NEIL3/PCNA/POLD1/POLE/POLE2

Table S4 Down-regulated KEGG pathways

ID	Description	Gene Ratio	P-value	Q-value	Gene ID
hsa04510	Focal adhesion	43/466	0.0000	0.0000	AKT3/BCL2/CAV1/CHAD/COL1A2/COL4A6/COL6A1/COL6A2/COL6A3/COL6A6/FLNA/FLNC/FLT1/FLT4/FYN/HGF/ILK/ITGA11/ITGA7/ITGA8/ITGA9/ITGB3/KDR/LAMA2/LAMA4/LAMB2/MAPK10/MYL9/MYLK/PARVA/PDGFC/PDGFD/PDGFRA/PDGFRB/PPP1R12B/PRKCA/RELN/THBS1/THBS4/TNXB/FIGF/VWF/ZYX
hsa04270	Vascular smooth muscle contraction	31/466	0.0000	0.0000	ACTA2/ACTG2/ADCY4/ADCY5/ADRA1A/ADRA1D/AGTR1/AVPR1A/CACNA1C/CALCRL/CALD1/EDNRA/GUCY1A2/ITPR1/KCNMA1/KCNMB1/KCNMB2/MRVI1/MYH11/MYL9/MYLK/NPR1/NPR2/PLA2G5/PPP1R12B/PPP1R14A/PRKCA/PRKG1/RAMP1/RAMP2/RAMP3
hsa04080	Neuroactive ligand-receptor interaction	50/466	0.0000	0.0000	ADCYAP1R1/ADRA1A/ADRA1D/ADRA2A/ADRA2C/ADRB3/AGTR1/AGTR2/AVPR1A/AVPR2/BRS3/CALCRL/CHRM2/CTSG/EDNRA/EDNRB/GH1/GLP2R/GRIA2/GRIA3/GRID1/GRIK5/GRM7/HTR1E/HTR2A/HTR2B/HTR4/LEPR/LPAR4/MAS1/MCHR1/NPY1R/NPY5R/P2RX1/PRL/PRLHR/PRLR/PTGER2/PTGER3/PTGFR/PTH1R/S1PR1/S1PR2/S1PR3/SSTR3/TACR1/TACR2/TBXA2R/THRA/VIPR2
hsa04020	Calcium signaling pathway	37/466	0.0000	0.0000	ADCY4/ADRA1A/ADRA1D/ADRB3/AGTR1/ATP2B4/AVPR1A/CACNA1C/CACNA1G/CACNA1H/CAMK2A/CHRM2/EDNRA/EDNRB/GNA14/HTR2A/HTR2B/HTR4/ITPKB/ITPR1/MYLK/NOS3/P2RX1/PDE1A/PDE1B/PDE1C/PDGFRA/PDGFRB/PLN/PRKCA/PTGER3/PTGFR/RYR3/SLC8A1/TACR1/TACR2/TBXA2R
hsa04022	cGMP-PKG signaling pathway	34/466	0.0000	0.0000	ADCY4/ADCY5/ADRA1A/ADRA1D/ADRA2A/ADRA2C/ADRB3/AGTR1/AKT3/ATP1A2/ATP1B2/ATP2B4/CACNA1C/EDNRA/EDNRB/GUCY1A2/ITPR1/KCNJ8/KCNMA1/KCNMB1/KCNMB2/MEF2C/MRVI1/MYL9/MYLK/NFATC4/NOS3/NPR1/NPR2/PDE2A/PLN/PRKG1/RGS2

					/SLC8A1
hsa04151	PI3K-Akt signaling pathway	51/466	0.0000	0.0000	AKT3/ANGPT1/ANGPT4/BCL2/CHAD/CHRM2/COL1A2/COL4A6/COL6A1/COL6A2/COL6A3/COL6A6/EPO/FGF10/FGF13/FGF2/FGF7/FGFR1/FLT1/FLT4/GH1/GNG11/GNG7/HGF/ITGA11/ITGA7/ITGA8/ITGA9/ITGB3/KDR/LAMA2/LAMA4/LAMB2/LPAR4/NGF/NOS3/NR4A1/PDGFC/PDGFD/PDGFRA/PDGFRB/PRKCA/PRL/PRLR/RELN/TEK/THBS1/THBS4/TNXB/FIGF/VWF
hsa04512	ECM-receptor interaction	20/466	0.0000	0.0000	CHAD/COL1A2/COL4A6/COL6A1/COL6A2/COL6A3/COL6A6/ITGA11/ITGA7/ITGA8/ITGA9/ITGB3/LAMA2/LAMA4/LAMB2/RELN/THBS1/THBS4/TNXB/VWF
hsa05414	Dilated cardiomyopathy	20/466	0.0000	0.0000	ACTC1/ADCY4/ADCY5/CACNA1C/CACNB2/DES/DMD/ITGA11/ITGA7/ITGA8/ITGA9/ITGB3/LAMA2/MYL3/PLN/SGCA/SGCD/SLC8A1/TPM1/TPM2
hsa04924	Renin secretion	16/466	0.0000	0.0001	ADCY5/ADCYAP1/ADCYAP1R1/ADRB3/AGTR1/AQP1/CACNA1C/EDNRA/GUCY1A2/ITPR1/KCNMA1/NPR1/PDE1A/PDE1B/PDE1C/PTGER2
hsa04261	Adrenergic signaling in cardiomyocytes	26/466	0.0000	0.0001	ACTC1/ADCY4/ADCY5/ADRA1A/ADRA1D/AGTR1/AGTR2/AKT3/ATP1A2/ATP1B2/ATP2B4/BCL2/CACNA1C/CACNB2/CAMK2A/MYL3/PLN/PPP1R1A/PRKCA/RAPGEF4/SCN1B/SCN4B/SCN7A/SLC8A1/TPM1/TPM2
hsa04911	Insulin secretion	18/466	0.0000	0.0001	ABCC8/ADCY4/ADCY5/ADCYAP1/ADCYAP1R1/ATP1A2/ATP1B2/CACNA1C/CAMK2A/KCNMA1/KCNMB1/KCNMB2/KCNN2/KCNN3/PRKCA/RAPGEF4/SNAP25/VAMP2
hsa05410	Hypertrophic cardiomyopathy (HCM)	17/466	0.0000	0.0003	ACTC1/CACNA1C/CACNB2/DES/DMD/ITGA11/ITGA7/ITGA8/ITGA9/ITGB3/LAMA2/MYL3/SGCA/SGCD/SLC8A1/TPM1/TPM2
hsa05412	Arrhythmogenic right ventricular cardiomyopathy (ARVC)	15/466	0.0000	0.0007	ACTN2/CACNA1C/CACNB2/CTNNA3/DES/DMD/ITGA11/ITGA7/ITGA8/ITGA9/ITGB3/LAMA2/SGCA/SGCD/SLC8A1

hsa05205	Proteoglycans in cancer	29/466	0.0000	0.0007	AKT3/ANK2/CAMK2A/CAV1/DCN/ESR1/FGF2/FGFR1/FLNA/FLNC/FZD4/HGF/HPSE2/HSPB2/IGF2/ITGB3/ITPR1/KDR/LUM/MMP2/PPP1R12B/PRKCA/SDC2/THBS1/TIMP3/TWIST1/TWIST2/WNT2/WNT9B
hsa04015	Rap1 signaling pathway	29/466	0.0001	0.0013	ADCY4/ADCY5/AKT3/ANGPT1/ANGPT4/FGF10/FGF13/FGF2/FGF7/FGFR1/FLT1/FLT4/GNAO1/HGF/ITGB3/KDR/LPAR4/MAGI2/NGF/PDGFC/PDGFD/PDGFRA/PDGFRB/PRKCA/RAPGEF4/RASGRP2/TEK/THBS1/FIGF
hsa04713	Circadian entrainment	17/466	0.0001	0.0016	ADCY4/ADCY5/ADCYAP1R1/CACNA1C/CACNA1G/CACNA1H/CAMK2A/GNAO1/GNG11/GNG7/GRIA2/GRIA3/GUCY1A2/ITPR1/PRKCA/PRKG1/RYR3
hsa04514	Cell adhesion molecules (CAMs)	22/466	0.0001	0.0018	CADM3/CD34/CDH5/CLDN5/CNTNAP1/ESAM/ICAM2/ITGA8/ITGA9/JAM2/JAM3/LRRC4B/MAG/NCAM1/NEGR1/NLGN3/NRXN2/NRXN3/PECAM1/PTPRM/SDC2/SELP
hsa04970	Salivary secretion	16/466	0.0002	0.0021	ADCY4/ADCY5/ADRA1A/ADRA1D/ADRB3/ATP1A2/ATP1B2/ATP2B4/BST1/GUCY1A2/ITPR1/KCNMA1/PRKCA/PRKG1/RYR3/VAMP2
hsa05200	Pathways in cancer	44/466	0.0003	0.0030	ADCY4/ADCY5/AGTR1/AKT3/AR/AXIN2/BCL2/COL4A6/CTNNA3/CXCL12/EDNRA/EDNRB/FGF10/FGF13/FGF2/FGF7/FGFR1/FOXO1/FZD4/GLI1/GNG11/GNG7/HGF/LAMA2/LAMA4/LAMB2/LPAR4/MAPK10/MITF/MMP2/PDGFRA/PDGFRB/PRKCA/PTCH2/PTGER2/PTGER3/RASGRP2/RUNX1T1/STAT5B/TGFBR2/FIGF/WNT2/WNT9B/ZBTB16
hsa04072	Phospholipase D signaling pathway	21/466	0.0004	0.0036	ADCY4/ADCY5/AGTR1/AKT3/AVPR1A/AVPR2/DGKB/FYN/GAB2/GRM7/LPAR4/MS4A2/PDGFC/PDGFD/PDGFRA/PDGFRB/PPAP2A/PPAP2B/PRKCA/PTGFR/RAPGEF4
hsa04014	Ras signaling pathway	29/466	0.0004	0.0036	AKT3/ANGPT1/ANGPT4/FGF10/FGF13/FGF2/FGF7/FGFR1/FLT1/FLT4/GAB2/GNG11/GNG7/HGF/KDR/MAPK10/NGF/PDGFC/PDGFD/PDGFRA/PDGFRB/PLA2G5/PRKCA/RASA4/RASA4B/RASGRF2/

					RASGRP2/TEK/FIGF
hsa04614	Renin-angiotensin system	7/466	0.0004	0.0042	AGTR1/AGTR2/CMA1/CPA3/CTSG/ENPEP/MAS1
hsa04024	cAMP signaling pathway	26/466	0.0005	0.0042	ADCY4/ADCY5/ADCYAP1R1/AKT3/ATP1A2/ATP1B2/ATP2B4/CACNA1C/CAMK2A/CHRM2/EDNRA/FXYD1/GLI1/GRIA2/GRIA3/HTR1E/HTR4/MAPK10/MYL9/NPR1/NPY1R/PLN/PTGER2/PTGER3/RAPGEF4/VIPR2
hsa02010	ABC transporters	10/466	0.0005	0.0042	ABCA10/ABCA6/ABCA8/ABCA9/ABCB1/ABCB5/ABCC8/ABCC9/ABCD2/ABCG2
hsa05218	Melanoma	12/466	0.0018	0.0153	AKT3/FGF10/FGF13/FGF2/FGF7/FGFR1/HGF/MITF/PDGFC/PDGFD/PDGFRA/PDGFRB
hsa04933	AGE-RAGE signaling pathway in diabetic complications	15/466	0.0020	0.0163	AGTR1/AKT3/BCL2/COL1A2/COL3A1/COL4A6/EGR1/FOXO1/MAPK10/MMP2/NOS3/PRKCA/STAT5B/TGFBR2/FIGF
hsa04925	Aldosterone synthesis and secretion	13/466	0.0022	0.0169	ADCY4/ADCY5/AGTR1/CACNA1C/CACNA1G/CACNA1H/CAMK1/CAMK2A/ITPR1/NPR1/NR4A1/PDE2A/PRKCA
hsa04724	Glutamatergic synapse	16/466	0.0026	0.0197	ADCY4/ADCY5/CACNA1C/DLG4/GNAO1/GNG11/GNG7/GRIA2/GRIA3/GRIK5/GRM7/ITPR1/PRKCA/SHANK3/SLC1A7/TRPC1

Table S5 Up-regulated GSEA pathways

NAME	Size	ES	NES	NOM p-value	FDR q-value
KEGG_ DNA replication	36	0.8605	3.1050	0.0000	0.0000
KEGG_ Cell cycle	124	0.6606	3.0257	0.0000	0.0000
KEGG_ Base excision repair	35	0.7141	2.5420	0.0000	0.0000
KEGG_ Mismatch repair	23	0.7528	2.3964	0.0000	0.0000
KEGG_ Systemic lupus erythematosus	109	0.5416	2.3960	0.0000	0.0000
KEGG_ Homologous recombination	27	0.7291	2.3908	0.0000	0.0000
KEGG_ Nucleotide excision repair	44	0.6368	2.3785	0.0000	0.0000
KEGG_ P53 signalling pathway	66	0.5705	2.2898	0.0000	0.0000
KEGG_ Proteasome	43	0.6214	2.2817	0.0000	0.0000
KEGG_ Spliceosome	123	0.4627	2.0906	0.0000	0.0004
KEGG_ Pyrimidine metabolism	93	0.4729	2.0471	0.0000	0.0006
KEGG_ Aminoacyl TRNA biosynthesis	41	0.5508	2.0251	0.0000	0.0006
KEGG_ Steroid biosynthesis	16	0.6876	1.9574	0.0000	0.0017
KEGG_ One carbon pool by folate	17	0.6671	1.9326	0.0000	0.0025
KEGG_ Oocyte meiosis	107	0.4266	1.8932	0.0000	0.0041
KEGG_ Progesterone mediated oocyte maturation	83	0.4359	1.8765	0.0000	0.0045
KEGG_ Antigen processing and presentation	50	0.4820	1.8220	0.0024	0.0073
KEGG_ Glycolysis gluconeogenesis	57	0.4505	1.7825	0.0000	0.0112
KEGG_ Primary immunodeficiency	35	0.5102	1.7760	0.0044	0.0115
KEGG_ Non-homologous end joining	12	0.6760	1.7697	0.0084	0.0118
KEGG_ RNA degradation	55	0.4491	1.7403	0.0000	0.0154
KEGG_ Cytosolic DNA sensing pathway	42	0.4678	1.7170	0.0047	0.0185
KEGG_ Bladder cancer	40	0.4490	1.6409	0.0046	0.0357
KEGG_ Folate biosythesis	11	0.6127	1.5703	0.0314	0.0631

Table S6 Down-regulated GSEA pathways

NAME	Size	ES	NES	NOM p-value	FDR q-value
KEGG_ Ribosome	87	-0.6146	-2.4324	0.0000	0.0000
KEGG_ Vascular smooth muscle contraction	112	-0.5767	-2.4139	0.0000	0.0000
KEGG_ Focal adhesion	196	-0.4470	-2.0197	0.0000	0.0038
KEGG_ Neuroactive ligand-receptor interaction	240	-0.4275	-1.9753	0.0000	0.0054
KEGG_ Calcium signalling pathway	169	-0.4386	-1.9248	0.0000	0.0070
KEGG_ Cell adhesion molecules (CAMs)	109	-0.4646	-1.9331	0.0000	0.0074
KEGG_TGF-β signalling pathway	85	-0.4575	-1.8121	0.0000	0.0193
KEGG_ Dilated cardiomyopathy	87	-0.4580	-1.8185	0.0000	0.0203
KEGG_ Hypertrophic cardiomyopathy (HCM)	81	-0.4433	-1.7179	0.0017	0.0451
KEGG_ MAPK signalling pathway	256	-0.3608	-1.6794	0.0000	0.0581
KEGG_ ECM-receptor interaction	83	-0.4174	-1.6421	0.0000	0.0726
KEGG_ Gap junction	89	-0.4106	-1.6290	0.0032	0.0764
KEGG_ Complement and coagulation cascades	67	-0.4223	-1.5935	0.0085	0.0874
KEGG_ Glycophingolipid biosynthesis ganglio series	15	-0.5868	-1.6005	0.0251	0.0887
KEGG_ ABC transporters	43	-0.4525	-1.5815	0.0139	0.0899
KEGG_ Arrhythmogenic right ventricular cardiomyopathy (ARVC)	72	-0.4034	-1.5508	0.0081	0.1067

Table S7 KEGG pathway analysis of PPI modules

Term	Count	P-value	Genes
hsa04080: Neuroactive ligand-receptor interaction	17	0.0000	MCHR1, PTGER3, NPBWR1, LPAR3, NPY1R, NPY5R, S1PR2, S1PR3, S1PR1, SSTR3, CHRM2, SSTR1, CNR1, GRM7, GALR2, S1PR5, ADRA2C
hsa05322: Systemic lupus erythematosus	11	0.0000	HIST1H3J, HIST1H4L, HIST1H2BD, HIST1H2AG, HIST1H2AD, HIST1H2BH, HIST2H4A, HIST1H2BO, HIST1H2BM, HIST1H4B, HIST1H2BL, HIST1H2BJ, HIST1H2AI, HIST1H3C, HIST1H2AJ, HIST3H2BB, HIST1H3F, HIST1H2AL
hsa04512: ECM-receptor interaction	10	0.0000	LAMA2, LAMB3, LAMB2, LAMC3, COL6A3, ITGB6, COL6A2, COL6A1, LAMC2, CHAD
hsa04510: Focal adhesion	10	0.0000	LAMA2, LAMB3, LAMB2, LAMC3, COL6A3, ITGB6, COL6A2, COL6A1, LAMC2, CHAD
hsa04110: Cell cycle	9	0.0000	CCNE2, CDC6, CDC45, MCM7, TP53, PKMYT1, TTK, CDK6, MCM5
hsa04062: Chemokine signaling pathway	8	0.0000	ADCY4, ADCY7, CXCL5, CCR1, ADCY5, CXCR3, CCL16, CXCL10
hsa04114: Oocyte meiosis	6	0.0002	ADCY4, ADCY7, ADCY5, SGOL1, CDC20, ESPL1
hsa05200: Pathways in cancer	6	0.0139	LAMA2, E2F2, LAMB3, LAMB2, LAMC3, LAMC2
hsa04060: Cytokine-cytokine receptor interaction	5	0.0475	CXCL5, CCR1, CXCR3, CCL16, CXCL10
hsa04020: Calcium signaling pathway	4	0.0349	ADCY4, PTGER3, ADCY7, CHRM2
hsa04270: Vascular smooth muscle contraction	4	0.0056	EDNRA, AVPR1A, ADRA1B, ADRA1D
hsa04115: p53 signaling pathway	4	0.0079	CCNE2, RRM2, TP53, CDK6
hsa00230: Purine metabolism	4	0.0350	PDE7B, PDE2A, RRM2, PDE11A
hsa04914: Progesterone-mediated oocyte maturation	3	0.0356	ADCY4, ADCY7, ADCY5
hsa04540: Gap junction	3	0.0402	ADCY4, ADCY7, ADCY5
hsa05414: Dilated cardiomyopathy	3	0.0449	ADCY4, ADCY7, ADCY5

Supplementry Figure S1：

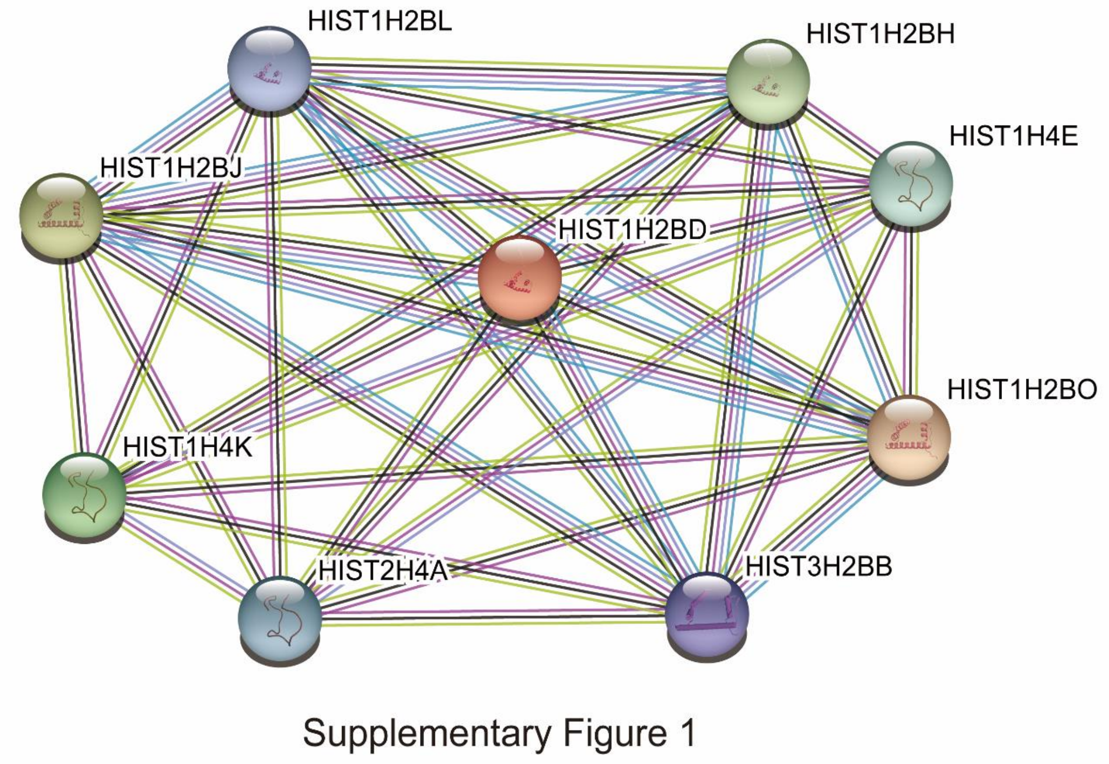

Protein-PPI network analysis of histone genes enriched in the SLE pathway. Each circle represents a node and each line represents an edge. The different color of the lines represents different types of interactions: light blue: curated databases; pink: experimentally determined; green: gene neighborhood; red: gene fusions; blue: gene co-occurrence; yellow: textmining; dark: co-expression; grey: protein homology.
